# The transitions and predictors of cognitive frailty with multi-state Markov model: a cohort study

**DOI:** 10.1186/s12877-022-03220-2

**Published:** 2022-07-01

**Authors:** Manqiong Yuan, Chuanhai Xu, Ya Fang

**Affiliations:** 1 State Key Laboratory of Molecular Vaccinology and Molecular Diagnostics, School of Public Health, Xiamen University, Xiamen, 361102 Fujian China; 2 Key Laboratory of Health Technology Assessment of Fujian Province, School of Public Health, Xiamen University, Xiamen, China

**Keywords:** Cognitive impairment, Physical frailty, Cognitive frailty, Multi-state Markov model

## Abstract

**Background:**

Cognitive frailty (CF) is characterized by the simultaneous presence of physical frailty and cognitive impairment. Previous studies have investigated its prevalence and impact on different adverse health-related outcomes. Few studies have focused on the progression and reversibility of CF and their potential predictors.

**Methods:**

Data were derived from the China Health and Retirement Longitudinal Study (CHARLS). A total of 4051 older adults with complete data on three waves of the survey (2011, 2013, and 2015) were included and categorized into four groups: normal state (NS), cognitive impairment (CI) only, physical frailty (PF) only and CF (with both PF and CI). A multi-state Markov model was constructed to explore the transitions and predicting factors of CF.

**Results:**

The incidence and improvement rates of CF were 1.70 and 11.90 per 100 person-years, respectively. The 1-year transition probability of progression to CF in those with CI was higher than that in the PF population (0.340 vs. 0.054), and those with CF were more likely to move to PF (0.208). Being female [hazard ratio (HR) = 1.46, 95%CI = 1.06, 2.02)], dissatisfied with life (HR = 4.94, 95%CI = 1.04, 23.61), had a history of falls (HR = 2.36, 95%CI = 1.02, 5.51), rural household registration (HR = 2.98, 95%CI = 1.61, 5.48), multimorbidity (HR = 2.17, 95%CI = 1.03, 4.59), and depression (HR = 1.75, 95%CI = 1.26, 2.45) increased the risk of progression to CF, whereas literacy (HR = 0.46, 95%CI = 0.33, 0.64) decreased such risk. Depression (HR = 0.43, 95%CI = 0.22, 0.84) reduced the likelihood of CF improvement, whereas literacy (HR = 2.23, 95%CI = 1.63, 3.07) increased such likelihood.

**Conclusions:**

Cognitive frailty is a dynamically changing condition in older adults. Possible interventions aimed at preventing the onset and facilitating the recovery of cognitive frailty should focus on improving cognitive function in older adults.

**Supplementary Information:**

The online version contains supplementary material available at 10.1186/s12877-022-03220-2.

## Background

Physical frailty (PF) is defined as a medical syndrome characterized by decrease of strength, endurance, and reduction of physiological functions that increase the vulnerability to stressors [1]. Both PF and cognitive impairment (CI) are common geriatric syndromes that increased the risk of adverse health-related outcomes, such as hospitalization, falls, dementia, and death [2–4]. These conditions are strongly associated and frequently coexist in the older people [5]. Previous studies have found that PF significantly increase the risk of CI [6] and vice versa [7], indicating that CI and PF may interact during aging [7, 8]. However, they are commonly regarded as two separate entities in traditional studies. A growing number of studies on their interrelationship has motivated the coining of the term “cognitive frailty” (CF). In 2013, the International Academy on Nutrition and Aging (I.A.N.A) and the International Association of Gerontology and Geriatrics (I.A.G.G) defined CF as a heterogeneous syndrome characterized by the simultaneous presence of both CI and PF without concurrent diagnosis of Alzheimer’s disease or other dementias [9]. Compared with a single syndrome (PF or CI), using CF as a predictor can improve the effectiveness of predicting the incidence of adverse health-related outcomes in older adults [10–12], indicating that it is critical to consider PF and CI as a complex phenotype to prevent adverse health-related outcomes. In that case, a key consideration in addressing CF is to find the potential predictors. Some studies have reported several factors associated with CF [13–15]. However, these findings mainly came from cross-sectional studies, which cannot establish the cause-and-effect relationships. Therefore, to determine the incidence and predictors of CF, longitudinal studies are essential to identify populations at risk and intensify public health intervention strategies.

Furthermore, the potential for reversibility may also characterize CF [9] as a possible target for early intervention to prevent CF-related adverse health outcomes, extend life span, and improve the quality of life in the CF population. Tang et al. observed that one in four individuals with CF at baseline reverted to the normal group while two reverted to only PF or mild cognitive impairment group after six years [16], confirming its modifiability under natural conditions. In that case, more studies are required to determine the magnitude of CF improvement and provide a clear description of transitions between CF, PF, and CI, and the influencing factors, using large-scale nationally representative data.

Most studies on CF have focused on the current status of its epidemic and its impact on adverse outcomes [17–21]. To our best knowledge, only one longitudinal study from Malaysia has reported the incidence of CF and its influencing factors [22]. It is critical to investigate this issue in different countries due to the differences in population characteristics(e.g., lifestyle, social environment, and mental health levels). More importantly, the view that CF is modifiable highlights the potential of targeting CF for promoting healthy aging. Determining the transitions between CI, PF, and CF, as well as possible predictors, on the one hand, could enable identification of high-risk stages prior to the onset of CF and interrupt its progression through early intervention to prevent incident CF and its related adverse health outcomes. On the other hand, it could help clinicians to manage patients more effectively. Unfortunately, to the best of our knowledge, such reports are currently unavailable.

To fill these gaps, the present longitudinal cohort study tried to estimate transition probabilities between CI, PF, and CF, as well as possible predictors of the aforementioned transitions using a continuous-time multi-state Markov model.

## Materials and methods

### Study population

The China Health and Retirement Longitudinal Study (CHARLS) was a nationally representative study of Chinese adults ≥ 45 years of age. The baseline survey was conducted in 2011, and a probability-proportional-to-size sampling approach was used to select 17,705 adults from 150 counties across 28 provinces. Follow-up visits were performed every two years. The CHARLS was approved by the Ethical Review Committee of Beijing University, and all the respondents signed informed consent forms. The details of the CHARLS survey have been described elsewhere [23].

**Fig. 1:**
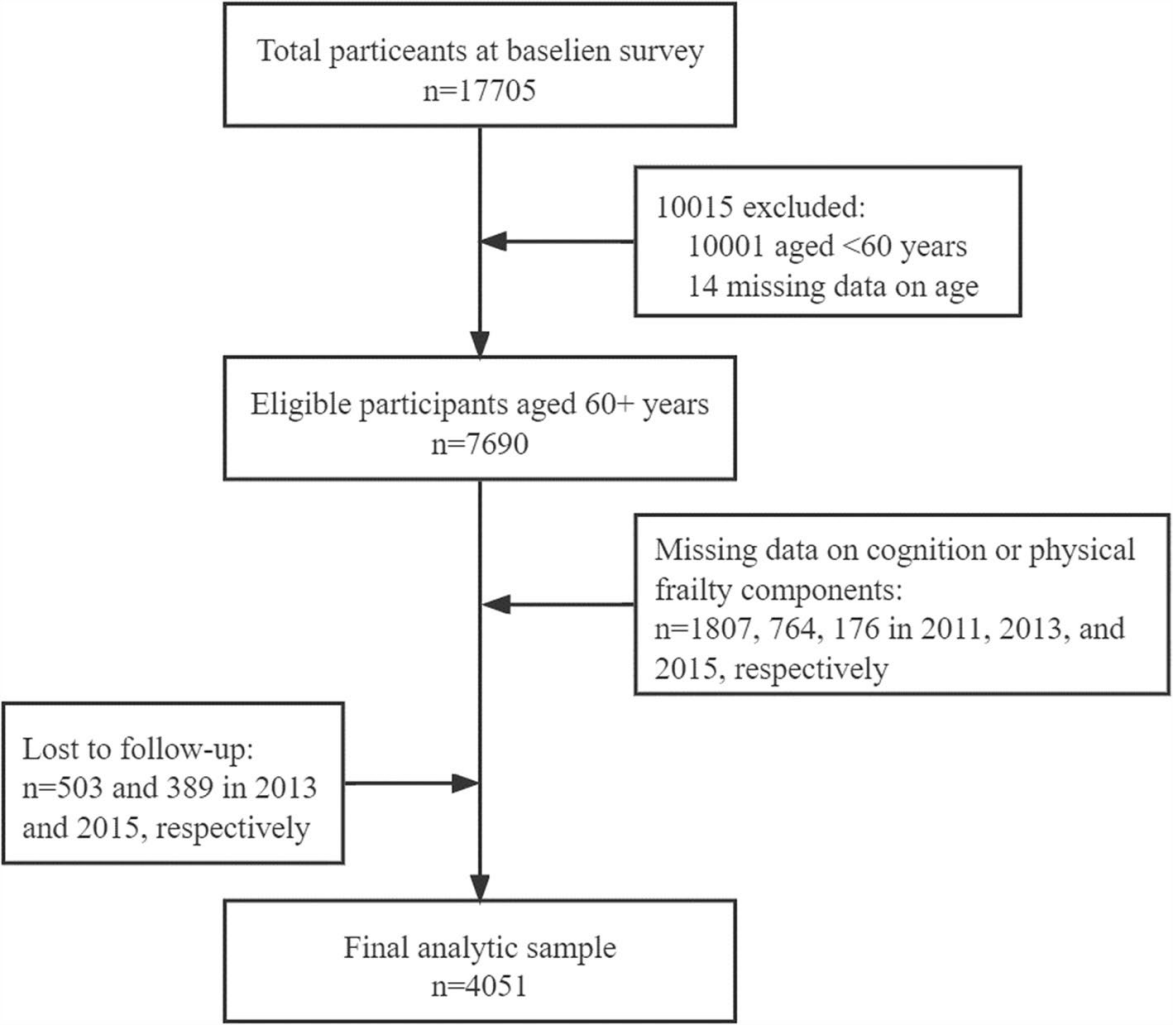
Flow chart of analytic sample

This study used data from the three waves (2011, 2013, and 2015) of the CHARLS. Of the 17,705 participants, we eliminated those < 60 years of age (*n* = 10,001) or those with missing information on age (*n* = 14), leaving 7690 eligible participants. During the follow-up period from 2011 to 2015, those with missing information on cognition or physical frailty components (*n* = 2747) or lost to follow-up (*n* = 892) were excluded. Finally, 4051 participants were included and evaluated in the current study. Figure 1 shows the selection process of the analytical sample.

### Measures

#### Cognitive impairment (CI)

Cognitive function was assessed by the Telephone Interview of Cognitive Status (TICS-10), word recall, and figure drawing, following previous publications that used data from the CHARLS study [24, 25]. The TICS-10 included date (year, month, and day), season, day of the week, and serial subtraction of 7 from 100(one point was awarded when the participants correctly calculated the result), for a total of 10 points. The word recall included immediate and delayed recall of 10 words (one point was awarded when a word was recalled correctly, and the final scores were calculated as the average of the two parts), for a total of 10 points. For figure drawing, the participants were shown a picture of two overlapped pentagons and were asked to draw a similar one. The participants were awarded 1 point if they could successfully complete the task; otherwise, they were given 0 point. The total score range of the three parts was 0–21, with higher scores indicating better performance [24, 25]. The participants were classified as having CI if their total score fell more than one standard deviation (SD) below age-appropriate norms [26, 27].

#### Physical frailty (PF)

PF was measured by a modified version of the Fried phenotype approach [2], which has been developed and validated in CHARLS [28]. It included five items: shrinking, weakness, slowness, low physical activity, and exhaustion. According to previous studies, the operationalized measurements used to define PF criteria were as follows: (1) Shrinking: it was defined as a subject’s body mass index (BMI) ≤ 18.5 kg/m^2^. (2) Weakness: it was defined using a handgrip strength test, and the cut-off points adjusted for gender and BMI. (3) Slowness: it was determined using a 2.5-m gait speed test with the cut-off values adjusted for gender and height. (4) Low physical activity: it was determined if participants reported that they did not walk for ≥ 10 min continuously in a usual week. (5) Exhaustion: it was determined using two items of the 10-item Center for Epidemiological Studies Depression Scale (CESD-10): “I felt everything I did was an effort” and “I could not get going”. If the subjects responded “sometimes or half of the time (3–4 days)” or “most of the time (5–7 days)” to either item, they were categorized as having self-reported exhaustion.

Scores were assigned to each physical frailty component (1 = present, 0 = absent), and the total scores were used to categorize the subjects as robust (score = 0), pre-physical frailty (score = 1–2), and physical frailty (score = 3–5). As proposed previously [22], subjects with pre-physical frailty and physical frailty were grouped together as one target group.

#### Cognitive frailty (CF)

Consistent with the definition by an (I.A.N.A/I.A.G.G) international consensus group [9], CF was defined as the simultaneity of both the CI and PF [9], and has been previously validated [29–31].

#### Normal state (NS)

The participants without PF and CI were categorized as NS.

#### Death state

The information on death was derived from the ‘Exit and Verbal autopsy questionnaire’ in 2013 and ‘Sample_Info.dta’ dataset in 2015.

#### Possible predictors

The predictors from the baseline measurement were divided into two subcategories: non-modifiable (age, gender, education [illiterate, literacy], and household registration [rural, non-rural]) and modifiable predictors.

The modifiable factors included multimorbidity, falls, injury, sleep duration, life-satisfaction, and depression. Regarding multimorbidity, the participants were asked, “Have you been diagnosed with the following conditions by a doctor?” and the conditions included hypertension, dyslipidemia, diabetes or high blood sugar, cancers or malignant tumors, chronic lung diseases, liver diseases, stroke, kidney diseases, stomach or other digestive diseases, emotional, nervous, or psychiatric problems, arthritis or rheumatism, asthma, and heart problems. Multimorbidity was defined as the presence of two or more of these conditions mentioned above. The history of falls was defined by asking the participants,“Have you fallen down in the last two years?”, with an answer of “Yes”. Injury history was defined by asking, “Have you ever been in a traffic accident or any other kind of major accidental injury and received medical treatment?,” with an answer of “Yes.” Sleep duration was obtained by self-reporting average hours per night during the past month, which was categorized as short (< 6 h), intermediate (6–9 h), and long (> 9 h) [32]. Life-satisfaction was a dichotomous variable with satisfied (including completely satisfied, very satisfied, and somewhat satisfied) and dissatisfied (containing not very satisfied and not at all satisfied). Depression was measured using the CESD-10 scale, with a score of ≥ 10 indicating depression [33, 34].

### Statistical analysis

Analyses were conducted using the software R, version 4.0.2, specifically the packages *msm* (multi-state modeling). The incidence rate was calculated as the number of new cases of CF in those without CF at baseline divided by the observed total person-years. Continuous and categorical variables were described as mean ± SD and counts, respectively. Analysis of variance (*ANOVA*) and chi-squared test were used to compare differences between the groups for continuous and categorical variables, respectively. The multi-state Markov analysis was conducted in two steps. First, the Markov model without covariates was performed to estimate the transition intensities and 1-year transition probabilities between states, as well as the mean sojourn times for each state. The second step was conducted to estimate the hazard ratio (HR) for covariates on transition intensities along with their 95% confidence interval. We used two-sided significance tests for all the analyses, with statistical significance set at *p* < 0.05.

### Multi-state Markov model

**Fig. 2:**
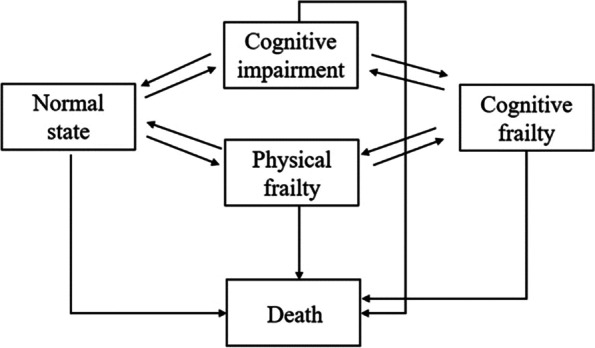
Schematic representation of five-state Markov model

A multi-state model describes how an individual moves between a series of states in continuous time, and fitting the multi-state Markov model to the panel data generally relies on the Markov assumption that future evolution only depends on the current state [35]. In this study, five interesting states were classified as NS, CI, PF, CF, and death, labelled 1, 2, 3, 4, and 5, respectively. The death state is an absorbing state, and other states are transient. With reference to previous studies [36, 37], direct transition were not permitted between non-adjacent states because the two events would not happen at exactly the same moment if investigated were continuously performed. Therefore, we assumed that the transition intensities between NS and CF, and between PF and CI were equal to zero in our continuous-time Markov model. Figure 2 presents the schematic representation of the proposed model and possible transitions.

### Maximum likelihood estimation of parameters

#### Transition intensity

Suppose an individual is in state *r* at time *t*. The movement on the discrete state space S = {1,2,3,4,5} is governed by transition intensities *q*_*rs*_ (*r*,* s* = 1, 2, 3, 4, 5), representing the instantaneous risk of moving from state *r* to state *s.* For example, *q*_*12*_ represents the instantaneous risk of moving from state 1(NS) to state 2(CI) in this study:

$$\underset{\delta t\to 0}{{q}_{rs}\left(t\right)=lim}P\left(S\left(t+\delta t\right)=\left.s\right|S\left(t\right)=r\right)/\delta t$$

Since it is not allowed directly transition between state 1 and state 4, and between state 2 and state 3, the transition intensity *q*_*14*_ = *q*_*41*_ = *q*_*23*_ = *q*_*32*_ = 0. The corresponding five-state transition intensity matrix can be defined as:

$$Q=\left(\begin{array}{ccccc}\text{-(q}{12}+ \text{q} {13}\text{+}{\text{q}}{15}\text{)}& {\text{q}}{12}& {\text{q}}{13}& {0}& {\text{q}}{15}\\ {\text{q}}{21}& \text{-(q}{21}+ \text{q} {24}\text{+}{\text{q}}{25}\text{)}& {0}& {\text{q}}{24}& {\text{q}}{25}\\ {\text{q}}{31}& {0}& \text{-(q}{31}+ \text{q} {34}\text{+}{\text{q}}{35}\text{)}& {\text{q}}{34}& {\text{q}}{35}\\ {0}& {\text{q}}{42}& {\text{q}}{43}& \text{-(q}{42}+ \text{q} {43}\text{+}{\text{q}}{45}\text{)}& {\text{q}}{45}\\ 0& 0& 0& 0& 0\end{array}\right)$$

#### Transition probability

Transition probability *P*_*rs*_*(t, t* + *u)* is the probability of being in state *s* at time *t* + *u,* given the state at time *t* is *r*. It was calculated in terms of *Q* using the Kolmogorov differential equations [38]. In this study, we estimated the 1-year transition probabilities between pair of states.

#### Mean sojourn time

The mean sojourn time describes the average period in a single stay in a state, which has an exponentially-distributed with mean -1/*q*_*rr*_. Here we estimated the mean sojourn time for each state except death state.

#### Goodness of fit tests

Diagnostic plots: By compared the observed and expected prevalence of each state under the model at a series of time point to assess the goodness-of-fit of model.

Pearson-type goodness-of-fit test: A formal test of goodness-of-fit is to construct tables of observed and expected number of transitions, which analogous to the classical Pearson χ^2^ test for contingency tables.

#### Regression model for covariates

The effect of individuals-level covariates *x*_*ij*_ on the transition intensity for individual *i* at time *j* is modelled using proportional intensity model [39], replacing *q*_*rs*_ with:

$$q_{rs}\left(x_{ij}\right)=q_{rs}^{\left(0\right)}exp\left(\beta^T{}_{rs}x_{ij}\right)$$

*β*_*rs*_ is a set of regression coefficients corresponding to covariates *x*_*ij*_. In this study, the covariates *x*_*ij*_ represent the modifiable (e.g., depression, multimorbidity, etc.) and non-modifiable factors (e.g., age, sex, etc.) measured at baseline.

### Sensitivity analysis

In sensitivity analysis, we did not restrict the transition intensity from state 1 (NS) to state 4 (CF) to zero (i.e. *q*_*14*_ and *q*_*41*_ are not equal to zero).The likelihood ratio test (LRT) was used to compare nested models. If the LRT produces *p* > 0.05, then the model being compared to fits the data is not better than the base model. Furthermore, we also calculated 1-year transition probability matrix of the new model to observe the differences in transition probabilities.

## Results

Table 1 shows the baseline characteristics of the 4051 individuals. The mean age of participants was 66.91 ± 5.82 years. The majority were males with rural household registration, educated, and satisfied with their lives. Approximately two-fifths of participants had depression, and 46.28% had self-reported multimorbidity. There was a relatively low percentage of injuries and fall history, whereas over three-quarters of individuals had an intermediate sleep duration at night. The prevalence of CI, PF, and CF at baseline was 3.51%, 49.40% and 11.53%, respectively.

**Table 1:** Baseline characteristics of the sample

Baseline characteristics	Full sample (*N* = 4051)	NS (*N* = 1441)	CI (*N* = 142)	PF (*N* = 2001)	CF (*N* = 467)	*χ*^*2*^*/F*	*p*
**Non-modifiable factors**							
Age, mean ± SD	66.91 ± 5.82	66.13 ± 5.17	65.91 ± 4.99	67.57 ± 6.22	66.78 ± 5.87	18.71^a^	< 0.001
Female, %	1838(45.37)	583(40.46)	102(71.83)	831(41.53)	322(68.95)	170.82	< 0.001
Education (illiterate)	1160(28.63)	246(17.07)	105(73.94)	472(23.59)	337(72.16)	694.86	< 0.001
Household registration (rural)	3083(76.10)	941(65.30)	129(90.85)	1578(78.86)	435(93.15)	192.39	< 0.001
**Modifiable factors**							
Life-satisfaction (dissatisfied)	535(13.21)	87(6.04)	14(9.86)	316(15.79)	118(25.27)	136.94	< 0.001
Injury	399(9.85)	133(9.23)	11(7.75)	204(10.19)	51(10.92)	2.20	0.530
History of falls	731(18.04)	202(14.02)	18(12.68)	387(19.34)	124(26.55)	43.69	< 0.001
Sleep duration						133.02	< 0.001
Intermediate	3103(76.60)	1221(84.73)	102(71.83)	1492(74.56)	288(61.67)		
Short	161(3.97)	52(3.61)	12(8.45)	437(21.84)	25(5.35)		
Long	787(19.43)	168(11.66)	28(19.72)	72(3.60)	154(32.98)		
Depression	1592(39.30)	202(14.02)	40(28.17)	1031(51.52)	319(68.31)	683.57	< 0.001
Multimorbidity	1875(46.28)	566(39.28)	63(44.37)	1008(50.37)	238(50.96)	46.24	< 0.001

*NS* normal state, *CI* cognitive impairment, *PF* physical frailty, *CF* cognitive frailty

^a^ *F* statistic for *ANOVA* analysis

### The incidence and improvement rates of CF

**Fig. 3:**
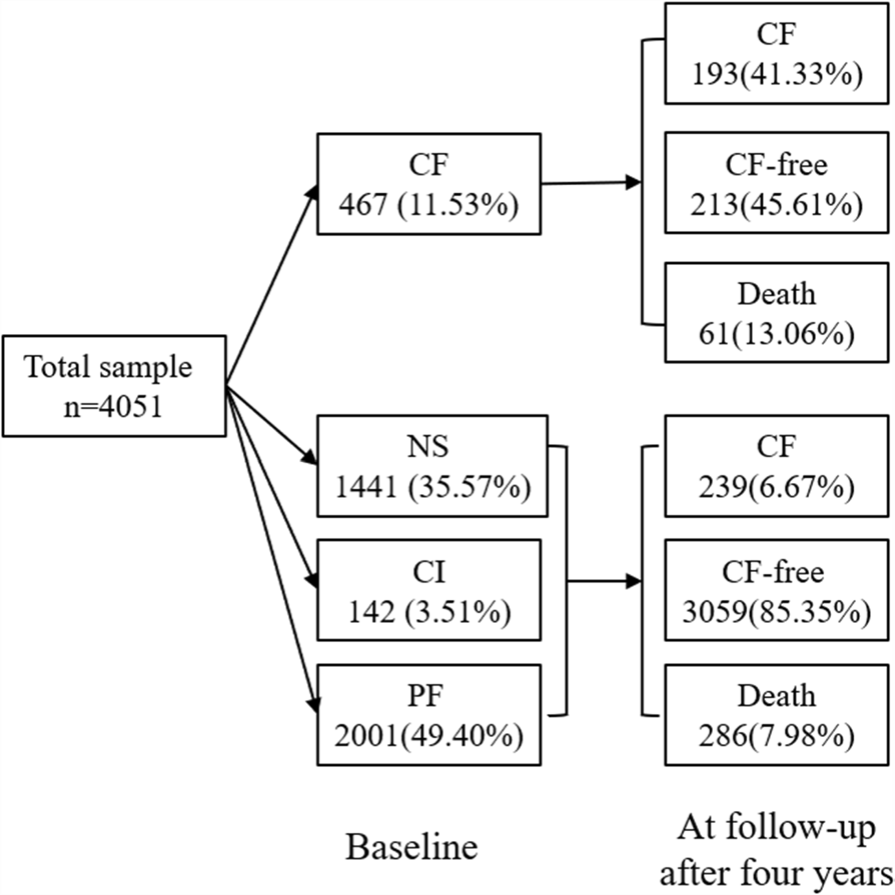
The distribution of states at follow-up after four years. *Note*: NS, normal state; CI, cognitive impairment; PF, physical frailty; CF, cognitive frailty; CF free, individuals are in state NS or CI or PF

Of the CF-free participants (individuals are in state NS or CI or PF) at baseline, 6.67% (*n* = 239) had CF at follow-up after four years (Fig. 3). The incidence rate of CF was 1.70/100 person-years. A total of 213 participants with CF at baseline moved to CF-free at follow-up after four years, representing approximately 45.61% of the cases. The observed improvement rate of CF was 11.90/100 person-years. In addition, there were 347 deaths during the follow-up, with 17.58% occurring in those with CF at baseline. The cumulative mortality was higher among those with CF than their counterparts (13.06% vs. 7.98%).

**Fig. 4:**
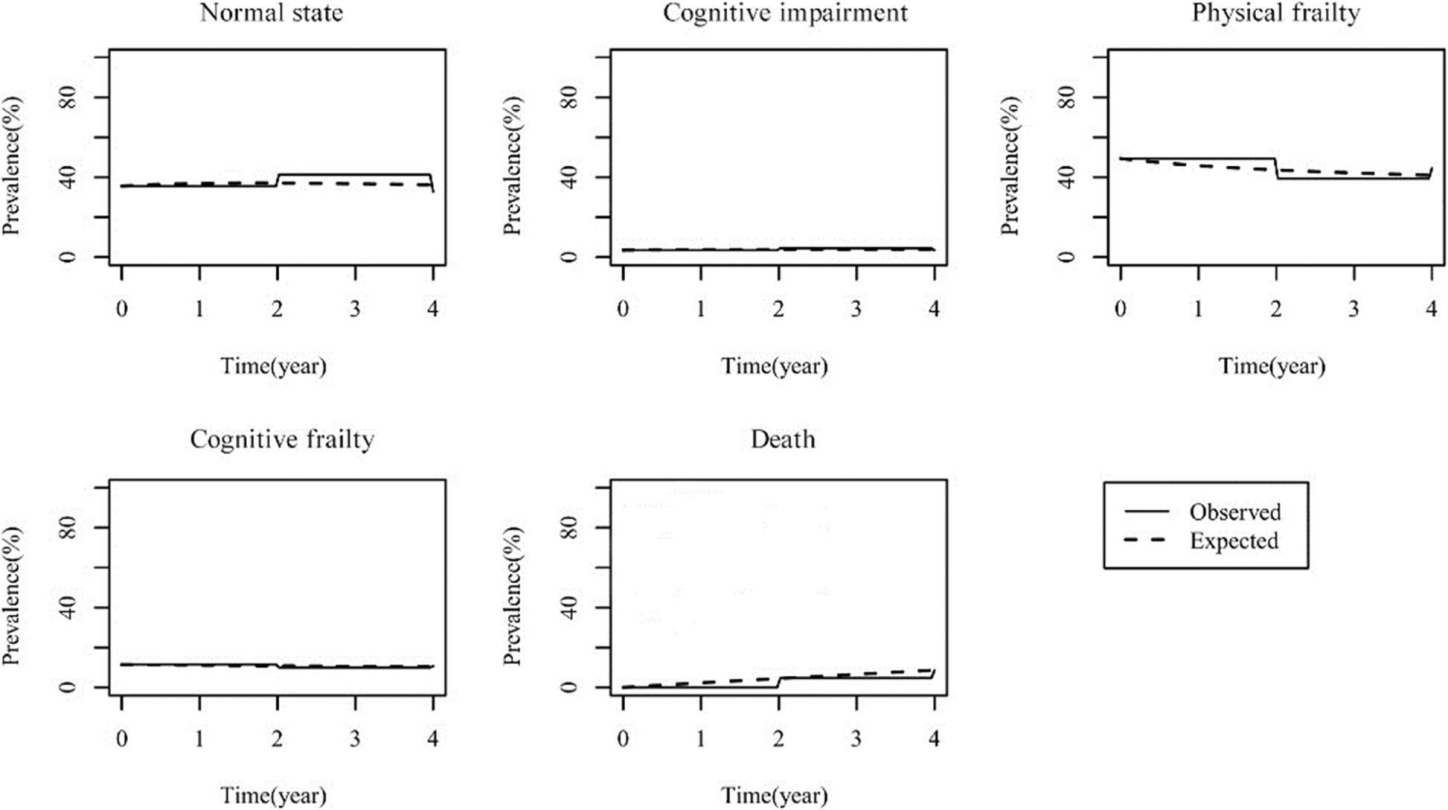
The compliance of each state observation rate with prediction rate

### Transition intensities and probabilities

Table 2 summarized the transition intensities and 1-year probabilities for each state. From the estimated intensity matrix, the transition from CI to CF was 8.7 times as likely to move from PF to CF. Moving from CF to PF was approximately 30% point higher than moving from CF to CI. From the transition probability matrix, we see NS individuals at baseline had a 70.18% probability of remaining at NS at year 1. In the case of transition, they were most likely to progress to the PF state. Those with CI only had a 38.79% probability of maintaining it and the likelihood of moving to CF was higher than moving to NS (33.95% vs.16.12%, respectively). Those with PF at baseline had a higher probability (69.23%) of staying there, and the probability of progressing to CF was lower than CI (5.37% vs. 33.95%). As for the CF population, the likelihood of transition to PF was 0.82 times higher than move to CI. Among the four groups at baseline, those with CF had the highest probability of death, followed by PF, CI, and NS.

**Table 2:** Transition intensities and probabilities for the multi-state Markov model

Transition	Intensities	1-year probability
NS-NS	-	0.702 (0.686, 0.716)
NS-CI	0.036 (0.028, 0.046)	0.019 (0.015, 0.024)
NS-PF	0.374 (0.346, 0.405)	0.251 (0.237, 0.266)
NS-CF	0	0.017 (0.015, 0.019)
NS-Death	0.008 (0.004, 0.014)	0.011 (0.009, 0.016)
CI-CI	-	0.388 (0.328, 0.442)
CI-NS	0.300 (0.228, 0.394)	0.161 (0.126, 0.201)
CI-PF	0	0.095 (0.083, 0.108)
CI-CF	0.756 (0.588, 0.972)	0.340 (0.286, 0.392)
CI-Death	0.005 (0.000, 0.082)	0.017 (0.012, 0.062)
PF-PF	-	0.692 (0.678, 0.706)
PF-NS	0.325 (0.301, 0.352)	0.218 (0.205, 0.231)
PF-CI	0	0.009 (0.008, 0.010)
PF-CF	0.086 (0.075, 0.098)	0.054 (0.048, 0.060)
PF-Death	0.030 (0.024, 0.036)	0.027 (0.023, 0.032)
CF-CF	-	0.582 (0.551, 0.611)
CF-NS	0	0.053 (0.048, 0.060)
CF-CI	0.254 (0.193, 0.335)	0.114 (0.093, 0.137)
CF-PF	0.333 (0.293, 0.379)	0.208 (0.187, 0.230)
CF-Death	0.051 (0.038, 0.068)	0.043 (0.034, 0.056)

The intervals are 95% confidence intervals

*NS* normal state, *CI* cognitive impairment, *PF* physical frailty, *CF* cognitive frailty

### Mean sojourn time for each state

The mean sojourn time of NS, CI, PF, and CF state in the present study were 2.39 ± 0.09, 0.94 ± 0.09, 2.27 ± 0.07, and 1.57 ± 0.11 years, respectively. The NS group had the longest mean sojourn time, while the CI had the shortest one.

### Model assessment

1. **Diagnostic plots**

Figure 4 specifically shows the observed and predicted prevalence of each state against time. As the figure depicted, the model fitted well.

(2) **Pearson-type goodness-of-fit test**

The test statistic was 102.02, and the *p* was 0.83, indicating that the model fitted well.

### The effect of covariates on transitions between CF, CI, and PF

To determine the effect of different modifiable and non-modifiable factors on the onset and transition of CF, first, we constructed a series of univariate multi-state Markov models to identify the influencing factors. Second, we performed a multivariate analysis to include variables that had been significant in the univariate models. Table 3 shows that being female (HR = 1.46, 95%CI = 1.06, 2.02), had a rural household registration (HR = 2.98, 95%CI = 1.61, 5.48), and depression (HR = 1.75, 95%CI = 1.26, 2.45) increased the risk of developing CF for those with PF, whereas literacy (HR = 0.46, 95%CI = 0.33, 0.64) decreased the risk. For CI individuals at baseline, the presence of multimorbidity (HR = 2.17, 95%CI = 1.03, 4.59), being dissatisfied with life (HR = 4.94, 95%CI = 1.04, 23.61) and had a history of falls (HR = 2.36, 95%CI = 1.02, 5.51) result in a higher risk of progression to CF. Literacy (HR = 2.23, 95%CI = 1.63, 3.07) increased the likelihood of CF improvement, whereas depression (HR = 0.43, 95%CI = 0.22, 0.84) reduced it.

**Table 3:** Hazard ratios (95%*CI*) for transition between CF, CI and PF by non-modifiable and modifiable factors

**Characteristic**	Progression from CI to CF	Progression from PF to CF	Improvement from CF to CI	Improvement from CF to PF
**Non-modifiable factors**				
Age (year, ref = 60–69)				
70–79	1.82 (0.67, 4.94)	1.14 (0.82, 1.59)	0.71 (0.23, 2.24)	0.99 (0.70, 1.40)
80-	0.72 (0.15,3.43)	1.18 (0.56, 2.52)	0.36 (0.08, 1.64)	2.17 (0.97, 4.85)
Gender ( ref = Male)				
Female	1.01 (0.52, 1.94)	1.46 (1.06, 2.02)	1.07 (0.54,2.15)	0.92 (0.66, 1.28)
Education (ref = Illiterate)				
Literacy	1.27 (0.54, 3.03)	0.46 (0.33, 0.64)	1.56 (0.68, 3.63)	2.23 (1.63, 3.07)
Household registration (ref = Non-rural)				
Rural	0.99 (0.45, 2.21)	2.98 (1.61, 5.48)	2.13 (0.54, 8.38)	0.82 (0.53, 1.28)
**Modifiable factors**				
Life-satisfaction (ref = Satisfied)				
Dissatisfied	4.94 (1.04, 23.61)	1.36 (0.94, 1.97)	3.68 (0.71, 19.21)	1.13 (0.78, 1.64)
Depression(ref = No)				
Yes	0.68 (0.34, 1.37)	1.75 (1.26, 2.45)	0.43 (0.22, 0.84)	1.39 (0.99, 1.96)
Multimorbidity(ref = No)				
Yes	2.17 (1.03, 4.59)	1.12 (0.82, 1.53)	1.97 (0.91, 4.27)	0.90 (0.67, 1.22)
Sleep duration (ref = Intermediate)				
Short	0.85 (0.34, 2.10)	1.72 (0.91, 3.26)	1.53 (0.60, 3.86)	1.21 (0.63, 2.36)
Long	1.95 (0.55, 6.95)	1.16 (0.85, 1.59)	1.99 (0.57, 6.98)	0.98 (0.71, 1.34)
History of falls ( ref = No)				
Yes	2.36 (1.02, 5.51)	1.20 (0.87, 1.65)	1.77 (0.74, 4.25)	1.01 (0.74, 1.41)

*CI* cognitive impairment, *PF* physical frailty, *CF* cognitive frailty

### Sensitivity analyses

In sensitivity analysis, The LRT results showed that the test statistic *G* was 0.61 and the degree of freedom was 2, with a *p* > 0.05, indicating no significantly differences between the two models. Furthermore, the differences in 1-year transition probabilities (Additional Table 1) between the two models were minor, indicating that the results were stable.

## Discussion

In this study, we longitudinally assessed the transitions between cognitive impairment, physical frailty, and cognitive frailty. Our findings confirmed the reversibility of cognitive frailty and showed the most likely direction of its transition. We found that people with CI had a higher probability of progressing to CF than that in PF, and those with CF were more likely move to PF than CI.

In the present study, the incidence rate of CF was 1.70/100 person-years, which is lower than that reported by Rivan et al. (7.10/100 person-years) [22], which may be attributed to differences in CF measurement tools, sample size (4051 vs. 282), and population, etc. Furthermore, the improvement rate of CF was 11.90/100 person-years. This is the first time, to our best knowledge, that a multi-state Markov model was applied to simultaneously study the onset and improvement in CF in a nationally representative population. The 1-year transition probability of progressing to CF in those with CI was higher than that in the PF population, indicating that early interventions aimed to prevent the onset of CF should focus on the phase of CI. The relationship between CI and PF may be reciprocal, but the magnitude of the mutual influence may vary. The view that poor cognition leads to PF is fairly evident and intuitive when treating patients with dementia and observing how physical disability develops concomitant CI [40]. Nevertheless, it is not clear whether PF results in cognitive problems. Several studies have shown that PF is associated with cognitive decline [10, 41]. Regarding individuals with CF, the finding that individuals with CF at baseline were more likely to reverse to PF at year 1 may partly be explained by the different reversal rates of CI and PF. A meta-analysis reported that the reversal rate of mild cognitive impairment was > 20%, whereas the improvement rate of PF was only 13.7%, which may be one of the reasons for the higher probability of transition from CF to PF.

Concerning the progression from CI to CF, individuals who were dissatisfied with their life, had a history of falls, and multimorbidity was associated with a higher risk of developing CF, consistent with previous studies [19, 20, 42]. PF and multimorbidity have been proved can mutually influence each other [43], with multimorbidity leading to the development of PF and eventually progressing to CF. Those reported being dissatisfied with their life were more likely to have a poor performance in social engagements [44], while increased social activity was an independent protective factor for CF in a previous study [45]. Previous studies demonstrated that PF was significantly associated with a higher risk of future falls [46], while the risk of falls was significantly higher in individuals with mild cognitive impairment [47, 48]. In the present study, there was a strong association between falls and CF in subjects with cognitive impairment, suggesting that fall avoidance might be advantageous to prevent CF.

Female gender, illiteracy, had a rural household registration, and depression were associated with a higher likelihood of progressing to CF in individuals with PF, consistent with prior studies [13, 20, 22, 49, 50]. Concerning modifiable factors, depression was confirmed by a longitudinal study as an independent risk predictor for CF [22]. The present study also showed that depression increased the risk of developing CF by 75% in subjects with PF. The co-existence of untreated depression and PF increased the risk of negative consequences at an older age, such as higher morbidity, lower quality of life, and accelerated CI [51], eventually leading to CF.

In terms of CF reversibility, individuals with CF and comorbid depression were 57% less likely to return to the CI state than those without depression. Previous studies showed that depression was associated with the onset of PF at different follow-up intervals [52, 53], reducing the likelihood of reverting to the PF status. The cognitive reserve hypothesis posits that education during early life prompts protective biological changes in the brain that can delay cognitive impairment [54]. One meta-analysis also reported that higher educational levels were associated with a high probability of reverting from mild cognitive impairment to a normal cognitive state [55], which is conducive to the transition from CF to PF.

The present study had several strengths. First, this is the first report on both the incidence of CF and transitions between CF, PF, and CF in Chinese older adults using longitudinal data. Second, we applied a multi-state Markov model, considered the best model for studying illness transitions [56] since it simultaneously takes into account all states, temporal information about inter-state transitions, and possible influencing factors. Third, the high quality of the data from a nationally representative population based study strengthen our findings. Nevertheless, this study also had several limitations. What should be noted is that individuals with missing data on CF measurement and lost to follow-up were excluded from the analysis; however, these individuals were more likely to be older and had higher illiteracy, weakening the extrapolation of our findings to a certain extent. Subsequent research may consider selecting a more representative sample or including dropped-out populations to enhance the generalizability of the results. Furthermore, information on multimorbidity and history of falls was self-reported by subjects, which could not be properly confirmed and might have affected the results to some extent. However, self-reported disease diagnosis has been widely used and shown to be valid in epidemiological studies [57]. Finally, since the assessment was not conducted continuously, information from the periods between follow-ups is not available, and the exact time of state transitions is unknown.

## Conclusion

This study demonstrated that cognitive impairment had a higher probability of progressing to cognitive frailty than physical frailty, and cognitive frailty were more likely to transit to physical frailty. Therefore, interventions aimed to preventing the onset and facilitating the recovery of cognitive frailty should be focus on improving cognitive function, thereby minimizing cognitive frailty related adverse health outcomes.

## Supplementary Information

**Additional file 1:  Additional Table 1.** Estimated1-year transition probability matrix of sensitivity analysis.

## Data Availability

The datasets analyzed during the current study was publicly available at http://charls.pku.edu.cn.

## Abbreviations

CI: Cognitive impairment
PF: Physical frailty
CF: Cognitive frailty
NS: Normal state
CHARLS: China Health and Retirement Longitudinal Study
I.A.N.A/I.A.G.G: International Academy on Nutrition and Aging/International Association of Gerontology and Geriatrics
TICS-10: Telephone Interview of Cognitive Status
BMI: Body mass index
CESD-10: The 10-item Center for Epidemiological Studies Depression Scale
LRT: Likelihood ratio test
HR: Hazard ratio
95%CI: 95% Confidence interval
