# Supplementary material for: The transitions and predictors of cognitive frailty with multi-state Markov model: a cohort study
Source: BMC Geriatr. 2022 Jul 1;22:550. doi: 10.1186/s12877-022-03220-2 (PMC9248089; doi:10.1186/s12877-022-03220-2)
Supplement: Supplementary file 1 — Additional file 1: Additional Table 1. Estimated1-year transition probability matrix of sensitivity analysis. [file 12877_2022_3220_MOESM1_ESM.docx]

**Additional Table 1 Estimated 1-year transition probability matrix of sensitivity analysis**

| Baseline  Status | 1-year transition probability | | | | |
| --- | --- | --- | --- | --- | --- |
|  | NS | CI | PF | CF | Death |
| NS | 0.702 | 0.019 | 0.251 | 0.017 | 0.011 |
| CI | 0.160 | 0.389 | 0.095 | 0.341 | 0.015 |
| PF | 0.218 | 0.009 | 0.692 | 0.054 | 0.027 |
| CF | 0.054 | 0.114 | 0.208 | 0.580 | 0.043 |

*Note*: NS, normal state; CI, cognitive impairment; PF, physical frailty; CF, cognitive frailty.
